# High Hydrostatic Pressure-Assisted Enzymatic Hydrolysis Affect Mealworm Allergenic Proteins

**DOI:** 10.3390/molecules25112685

**Published:** 2020-06-09

**Authors:** Abir Boukil, Véronique Perreault, Julien Chamberland, Samir Mezdour, Yves Pouliot, Alain Doyen

**Affiliations:** 1 Department of Food Sciences, Institute of Nutrition and Functional Foods (INAF), Université Laval, Quebec City, QC G1V 0A6, Canada; abir.boukil.1@ulaval.ca (A.B.); veronique.perreault.5@ulaval.ca (V.P.); julien.chamberland@fsaa.ulaval.ca (J.C.); Yves.Pouliot@fsaa.ulaval.ca (Y.P.); 2 AgroParisTech, UMR782 Paris-Saclay Food and Bioproduct Engineering (SayFood and Bioproduct Engineering), 1, rue des Olympiades, 91077 Massy, France; samir.mezdour@agroparistech.fr

**Keywords:** high hydrostatic pressure, mealworm proteins, enzymatic hydrolysis, allergenic proteins, proteomics analysis

## Abstract

Edible insects have garnered increased interest as alternative protein sources due to the world’s growing population. However, the allergenicity of specific insect proteins is a major concern for both industry and consumers. This preliminary study investigated the capacity of high hydrostatic pressure (HHP) coupled to enzymatic hydrolysis by Alcalase^®^ or pepsin in order to improve the in vitro digestion of mealworm proteins, specifically allergenic proteins. Pressurization was applied as pretreatment before in vitro digestion or, simultaneously, during hydrolysis. The degree of hydrolysis was compared between the different treatments and a mass spectrometry-based proteomic method was used to determine the efficiency of allergenic protein hydrolysis. Only the Alcalase^®^ hydrolysis under pressure improved the degree of hydrolysis of mealworm proteins. Moreover, the in vitro digestion of the main allergenic proteins was increased by pressurization conditions that were specifically coupled to pepsin hydrolysis. Consequently, HHP-assisted enzymatic hydrolysis represents an alternative strategy to conventional hydrolysis for generating a large amount of peptide originating from allergenic mealworm proteins, and for lowering their immunoreactivity, for food, nutraceutical, and pharmaceutical applications.

## 1. Introduction

Edible insects have garnered increased interest for human consumption due to their high nutritional value and low environmental impact when compared to conventional livestock. Moreover, edible insects were targeted as a potential alternative protein resource to address the problem of a global food crisis. In Western countries, mealworm (*Tenebrio molitor*) is of particular interest, being evidenced in recent years by several publications related to protein quality and the improvement of techno-functional properties [1,2,3]. However, recent studies described an allergenic risk that was related to the consumption of edible insects due to potential cross-reactivity with other arthropods, especially crustaceans [4,5,6]. Tropomyosin, arginine-kinase, but also other insect proteins, such as larval cuticle protein, myosin light, and heavy chain, as well as troponin, are potentially involved in allergenic reactions in cross-reactivity with other allergenic species (shrimp, prawn, and crab) [5,7,8]. Consequently, particularly for consumers with a food allergy to crustaceans, it is necessary to consider the risk of an allergic reaction after consuming edible insects.

Different food processing methods, such as boiling [9,10], autoclaving [11], extrusion [12], microwave [13], pulsed-electric field [14], ultrasound [15], and HHP [16,17], may alter the intrinsic structure of food proteins and, consequently, decrease their allergenic properties. These processes may be coupled to enzymatic hydrolysis to further decrease the allergenicity of a wide range of conventional food proteins and generate protein hydrolysates that could potentially be integrated into specific formulations for food-allergic patients [18]. Similar strategies for reducing protein allergenicity were applied to edible insect products. Specifically, Alcalase^®^ and pepsin were used to decrease the protein allergenicity of a wide range of food matrices, including edible insects. Indeed, Hall et al. [19] demonstrated that increasing the degree of hydrolysis (DH) of cricket proteins after proteolysis by Alcalase^®^ positively impacted their bioactive potential while lowering the reactivity to tropomyosin. Van Broekhoven, Bastiaan-Net, de Jong, and Wichers [5] showed that mealworm protein allergenicity decreased significantly after heat processing and in vitro digestion by pepsin. Additionally, the application of microwave-assisted enzymatic hydrolysis by Alcalase^®^ to cricket protein was an efficient method for generating hypoallergenic peptide fractions [20].

High hydrostatic pressure (HHP), a non-thermal technology that applies isostatic pressure from 100 to 1,000 MPa, induces the modification of secondary, tertiary, and quaternary structures of proteins, causing protein unfolding due to the rupture of noncovalent bonds (hydrogen, hydrophobic, and ionic bonds) [21]. The use of HHP combined with diverse enzymes to reduce protein allergenicity has been previously reviewed [18]. However, HHP-assisted enzymatic hydrolysis to modify allergenic protein digestion from edible insects has not yet been reported. Consequently, the aims of this preliminary work were 1) to apply HHP in combination with enzymatic hydrolysis by Alcalase^®^ or pepsin to generate protein hydrolysates from mealworm meal and 2) to evaluate different pressurization strategies (applied as a pretreatment before enzymatic hydrolysis or simultaneously with enzymatic hydrolysis) to potentially improve the in vitro digestion of mealworm allergenic proteins.

## 2. Materials and Methods

### 2.1. Raw Material and Preparation of Mealworm Samples

Mealworm (*Tenebrio molitor)* meal powder was purchased from Entomo Farms (Norwood, ON, Canada). The meal was suspended in a Tris-HCl solution (pH 7) at a concentration of 5% w/V and stirred for 60 min at room temperature. Finally, the insect suspension was stored overnight at 4 °C prior to enzymatic hydrolysis.

### 2.2. Proximate Composition of Mealworm Meals

The crude fat content was obtained after hexane extraction based on a Soxhlet method (AOAC 960.39). The moisture and ash contents were determined by the Association of Official Analytical Chemists (AOAC) methods 925.09 and 923.03, respectively. The crude protein content was obtained by the Dumas method (Rapid Micro N Cube, Elementar, Langenselbold, Germany) while using a protein-to-nitrogen conversion factor of 4.76 [22]. The chitin content was determined according to the method described by Spinelli et al. [23]. Proximate composition of mealworm meal on dry basis was 45.8 ± 0.2 protein, 15.5 ± 0.9 lipid, 5.2 ± 0.0 ash, and 7.7 ± 1.0 chitin.

### 2.3. High Hydrostatic Pressure-Assisted Enzymatic Hydrolysis of Mealworm Proteins

Enzymatic hydrolysis of mealworm proteins was performed prior to (pretreated) or during (simultaneous) pressurization while using a laboratory scale HHP system (Mini FoodLab FPG5620, Stansted Fluid Power LTD, Essex, UK) with a rate of pressurization of 50 MPa/min. The pressure system has a maximum capacity of 900 MPa and a glycol/water solution (30:70) was the pressure transfer fluid. Alcalase^®^ (Subtilisin from *Bacillus licheniformis*, Sigma Aldrich, St. Louis, MO, USA) and pepsin (BD Difco, Franklin Lakes, NJ, USA) were the enzymes used for hydrolysis. The pressure and duration parameters applied during enzymatic hydrolysis for both pretreated and simultaneous conditions were 380 MPa for 1 min. The specific value of 380 MPa was chosen because Zhang et al. [24] demonstrated a significant decrease of Alcalase^®^ activity when the pressure reached 400 MPa, which indicated that this level of pressurization induced partial denaturation of the enzyme. Similarly, Curl and Jansen [25] observed that the activity of pepsin that was retained when diluted in a buffer solution at pH 1.9 was close to 100% at 400 MPa, but decreased drastically at higher pressures.

For pretreatment experiments, the mealworm meal suspension was first pressure-treated at 380 MPa for 1 min and then hydrolyzed at atmospheric pressure (0.1 MPa) while using Alcalase^®^ or pepsin. The choice of these enzymes was based to their ability to hydrolyse the chitin-protein complex from arthropods and, consequently, to improve the recovery of separate protein and chitin separately, as described by Le Roux et al. [26] and De Holanda and Netto [27], respectively. Moreover, Alcalase^®^ and pepsin were already used to decrease protein allergenicity in edible insect matrices [5,19]. The hydrolysis parameters for Alcalase^®^ were an enzyme/substrate (E/S) ratio of 0.03% w/w at pH 8.5 and 60 °C for 120 min. The pepsin hydrolysis conditions were an E/S ratio of 0.25% w/w at pH 2.0 and 40 °C for 240 min. Hydrolysis durations of 120 and 240 min were determined as optimal for inducing the separation of the chitin-protein complexes in initial edible insect meals (results not shown) and consequently improved the recovery of protein/peptide fraction in the hydrolysates. For the simultaneous treatment condition, Alcalase^®^ or pepsin enzyme, at the same E/S ratio, pH, and temperature as the pretreated condition, were added to the insect meal suspensions (5% w/V) before pressurization. The mixture of mealworm suspension and enzyme was also pressure-treated at 380 MPa for 1 min. An external thermoregulation system was used to maintain the enzyme’s optimal temperature (60 and 40 °C for Alcalase^®^ and pepsin, respectively) during pressurization. At the end of pressure treatment, decompression was instantaneous and mealworm samples were recovered. The remaining duration of hydrolysis (total hydrolysis of 120 and 240 min for Alcalase^®^ and pepsin, respectively) was carried out at 0.1 MPa (atmospheric pressure) with constant pH and temperature control. The pH was controlled during control and pretreatment conditions, but not during simultaneous treatment (1 min), since it was not possible to open the reactor pressure vessel. However, it was controlled after pressure treatment and until the end of the hydrolysis. The control condition consisted of mealworm suspension (5% w/V) that was digested at atmospheric pressure (0.1 MPa) for 120 and 240 min with Alcalase^®^ and pepsin, respectively. During the hydrolysis step, and for all conditions (control, pretreated, and simultaneous digestions), a sample of mealworm hydrolysate was collected every 2 min for the first 10 min of hydrolysis and then every 30 min until the end of digestion. Samples that were collected during and at the end of hydrolysis were immediately immersed in a 90 °C water bath for 5 min to inactivate the enzyme and then centrifuged at 9000× *g* for 10 min. Supernatants, corresponding to soluble peptide fractions and potential non hydrolyzed soluble proteins, were recovered and stored at 5 °C until analysis.

### 2.4. Analysis

#### 2.4.1. Determination of the Degree of Hydrolysis

The degree of hydrolysis (DH), which is the proportion of peptide bonds released during in vitro protein digestion, was calculated according to the method that was described by Church et al. [28] for mealworm hydrolysates collected during and at the end of enzymatic hydrolysis, for control, pretreated, and simultaneous conditions. Briefly, 150 µL of mealworm hydrolysates (5% w/V) diluted by a factor of 60 was added to 3 mL of o-phthaldialdehyde (OPA) reagent. The mixture was incubated at room temperature for 2 min, transferred to polystyrene cuvettes and the absorbance at 340 nm measured in an Agilent 8453 UV-Visible spectrophotometer (Agilent Technologies, Santa Clara, CA, USA). DL-leucine was used as a standard with concentrations ranging from 0.75 mM to 3 mM. All of the samples were analyzed in triplicate. The DH was calculated using Equation (1):

(1) $$\mathrm{DH}\;(\%)=\left[\frac{h}{htot}\right]\times 100$$

where *h_tot_* of mealworm was determined from the amino acid composition of the protein, as the sum of mmols of the individual amino acids per g. The *h_tot_* used in this study was 8.64 meq/g. The values of (*h*) were obtained by reference to a standard curve of absorbance at 340 nm versus mg/L amino nitrogen (using L-leucine) [29].

#### 2.4.2. Digestion Profiles of Mealworm Proteins

The degradation profiles of the mealworm proteins after both pressurization conditions (pretreated and simultaneous) were obtained by sodium dodecyl sulfate-polyacrylamide gel electrophoresis (SDS-PAGE) under reducing conditions and compared to the control samples (enzymatic hydrolysis without pressurization treatment). Fifteen microliters of sample from initial and final hydrolysates (t = 0, 120 and 240 min) of both insect species was first diluted in 50 µL of deionized water. A volume of 25 µL of sample buffer (5% 2-mercaptoethanol, 95% Laemmli buffer) (Bio-Rad, Mississauga, ON, Canada) was added to 25 µL of each diluted sample. Subsequently, the solutions (hydrolysate and sample buffer) were immersed in a boiling water bath for 10 min and cooled on ice before injecting 10 µL per well. Electrophoresis was performed using 4–20% TGX Stain-Free polyacrylamide gel (Bio-Rad, Mississauga, ON, Canada) at 15 mA for 1 h at room temperature. The proteins were then stained with Coomassie blue (1 g/L of Coomassie Brilliant Blue R-250, 10% acetic acid, 40% ethanol, and 50% water) and destained with a solution of 10% V/V methanol and 10% V/V acetic acid. The MW of insect proteins and peptides were estimated using MW markers (Precision Plus Protein™ 161-0373 All Blue Prestained Protein Standards, Bio-Rad, Mississauga, ON, Canada). Images of the gels were captured using the ChemiDoc™ MP Imaging System (ChemiDoc MP, Bio-Rad, Mississauga, ON, Canada).

#### 2.4.3. Protein Identification by Mass Spectrometry

Mass spectrometry (MS) analysis was performed by the Proteomics Platform of the Research Center of the *Centre Hospitalier Universitaire* (CHU) of Quebec City (QC, Canada). First, final hydrolysates (120 or 240 min of hydrolysis) generated by Alcalase^®^ and pepsin in vitro digestion for each condition (control, pretreatment, and simultaneous conditions) were desalted on an Oasis HLB column (Waters, Mississauga, ON, Canada) and the peptides were quantified at 205 nm while using a nanodrop (ThermoFisher, Waltham, MA, USA). Peptide samples (1 µg) were analyzed by Liquid Chromatography-Mass Spectrometry (LC-MS/MS) on an Ekspert NanoLC425 (Eksigent, Redwood City, CA, USA) coupled to a 5600+ Triple TOF mass spectrometer (Sciex, Framingham, MA, USA) that was equipped with a nanoelectrospray ion source. The peptides were trapped on a pepmap 5 mm × 0.3 mm (ThermoFisher, Waltham, MA, USA) cartridge at 4 µL/min then separated on a self-packed picofrit column (New Objective, Woburn, MA, USA) with Reprosil 3 µL, (120A C18, 15 cm × 0.075 mm internal diameter), (Dr Maisch, Ammerbuch, Germany). The peptides were eluted with a linear gradient from 8–35% solvent B (acetonitrile, 0.1% V/V formic acid) in 30 min, at 300 ηL/min. Mass spectra were acquired using a data dependent acquisition mode and Analyst software version 1.7 (Sciex, Framingham, MA, USA). Each full scan mass spectrum (400 to 1,250 m/z) was followed by collision-induced dissociation of the twenty most intense ions. Dynamic exclusion was set for a period of 20 s and a tolerance of 100 ppm.

Mascot Generic Format (MGF) peak list files were created using Protein Pilot software (version 4.5, Sciex, Concord, ON, Canada). The MGF sample files were then analyzed using Mascot software (version 2.5.1, Matrix Science, London, UK). Uniprot databases were used to detect contaminants and align protein sequences while using the *Tenebrionidae* family (24,496 entries) database, assuming no enzyme. A fragment ion mass tolerance of 0.100 Da and a parent ion tolerance of 0.100 Da were used. The deamidation of asparagine and glutamine, and oxidation of methionine, were specified in Mascot as variable modifications.

Scaffold software (version 4.8.4, Proteome Software Inc., Portland, OR, USA) was used to validate MS/MS-based peptide and protein identifications. Peptide identifications were accepted if they could be established at a probability greater than 95.0% by the Scaffold Local False Discovery Rate (FDR) algorithm. Protein identifications were also accepted if they could be established at a probability that is greater than 95.0% and if they contained at least two identified peptides. Protein probabilities were assigned by the Protein Prophet algorithm [30]. Proteins that contained similar peptides that could not be differentiated based on MS/MS analysis alone were grouped to satisfy the principles of parsimony.

### 2.5. Statistical Analysis

The pressurization assays, enzymatic hydrolysis, and proximate composition were performed in triplicate. The DH results were expressed as mean ± standard deviation (SD). These data were subjected to a one-way analysis of variance (ANOVA) using Sigma Plot 14.0 (Systat Software Inc., Chicago, IL, USA). A 95% confidence level was used for all analyses.

## 3. Results

### 3.1. Mealworm Protein Degradation during Enzymatic Hydrolysis

**Figure 1:**
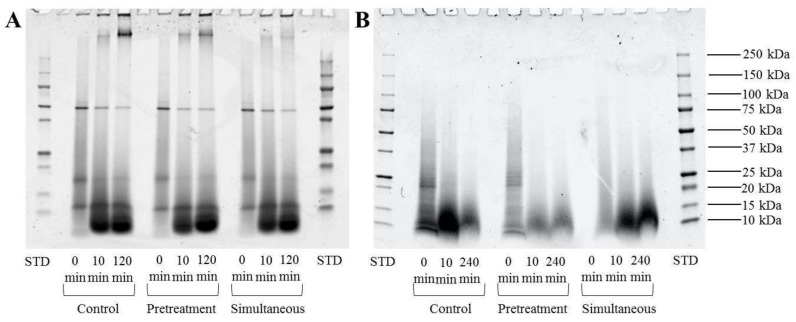
Protein degradation of mealworm proteins during enzymatic hydrolysis by Alcalase^®^ (**A**) and pepsin (**B**) for control (0.1 MPa) and pressurization conditions (pretreated and simultaneous at 380 MPa for 1 min).

Figure 1 presents the degradation of mealworm soluble proteins that were recovered at the beginning, during, and at the end of the enzymatic hydrolysis for control and pressurized conditions (pretreated and simultaneous). Regarding protein profiles during Alcalase^®^ hydrolysis (Figure 1A), a band, whose intensity increased as a function of hydrolysis duration, was detected in wells for control, pretreated, and simultaneous conditions. Whatever the condition, three distinctive bands that corresponded to proteins with molecular weights (MW) close to 10, 20, and 50 kDa were observed. The protein degradation profiles for hydrolysates after 10 and 120 min of Alcalase^®^ hydrolysis for control, pretreated, and simultaneous conditions were similar. More specifically, the same bands, as listed for control at t = 0 min (10, 20, and 50 kDa), were detected, but heavy bands close to 10 kDa were observed after 10 and 120 min of hydrolysis, resulting in mostly peptide fragments. A band with MW between 150 and 250 kDa observed after 10 min of hydrolysis, and whose intensity was increasing at 120 min of hydrolysis, was detected for control and pretreatment conditions but was absent at t = 0 min. When compared to Alcalase^®^, non-distinctive bands were observed for the pepsin control at t = 0 min except for the band at 20 kDa (Figure 1B). However, while peptides with molecular weights close to 10 kDa MW were detected for all conditions, the bands were more intense for simultaneous conditions after 10 and 240 min of hydrolysis as compared to the pretreatment condition. Under the control conditions, peptides with molecular weight close to 10 kDa MW were more concentrated after 10 min of hydrolysis as compared to 240 min.

### 3.2. Effect of High Hydrostatic Pressure Coupled to Enzymatic Digestion on the Degree of Hydrolysis of Mealworm Proteins

**Figure 2:**
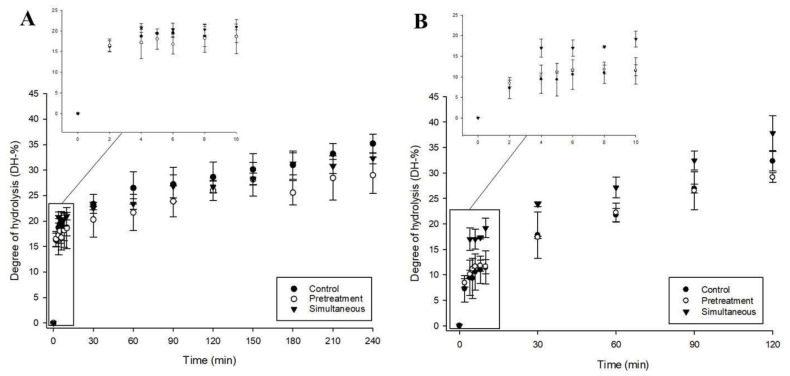
Degree of hydrolysis (%) of mealworm proteins digested by Alcalase^®^ (**A**) and pepsin (**B**) for control (0.1 MPa), pretreated, and simultaneous conditions (380 MPa) (n = 3 ± SD).

Figure 2 shows the evolution of DH for mealworm proteins digested by Alcalase^®^ and pepsin under three different conditions: without pressure (control), HHP applied as a pretreatment before hydrolysis, and HHP applied simultaneously during in vitro digestion. The degree of hydrolysis during in vitro digestion of mealworm protein by Alcalase^®^ increased rapidly from 0 to 10 min and linearly until the end of hydrolysis (120 min), no matter the condition (control, pretreatment, and simultaneous conditions) (Figure 2A). The evolution of DH was similar for the control and pretreatment conditions with respective final values of 32.29 ± 1.90%, 37.82 ± 3.43%, and 29.12 ± 0.95% (p>0.05). However, the degree of hydrolysis under HHP (simultaneous condition) was higher than control (21.75 ± 1.27%) and pretreated (22.20 ± 1.89%) conditions up to 60 min (*p* < 0.05) (27.15 ± 2.02%), but remained similar from 60 to 120 min (*p* > 0.05).

The evolution of the DH during mealworm protein hydrolysis by pepsin was similar to that of Alcalase^®^, with values of 35.20 ± 1.87%, 29.01 ± 3.55%, and 32.31 ± 1.09% for the control, pretreated, and simultaneous conditions, respectively (p>0.05) (Figure 2B). When compared to hydrolysis by Alcalase^®^, the DH increased steadily over time and was lower at the end of hydrolysis (120 min for Alcalase^®^ and 240 min for pepsin).

### 3.3. Determination of Mealworm Allergenic Protein Precursors from Hydrolysates

Table 1 presents the determination of mealworm allergenic protein precursors from peptides generated after 120 min of Alcalase^®^ hydrolysis or 240 min of pepsin hydrolysis in vitro digestion. The total spectrum count (TSC) value, defined as the total number of spectra identified for a protein, is of particular interest, since this parameter is a semi-quantitative measure for a given protein abundance in proteomic studies [31]. Indeed, previous studies have demonstrated that the spectral counts of proteins correlate linearly with protein abundances in complex samples [32,33,34]. High throughput mass spectrometry identified a total of 110 proteins and close to 2800 spectrum counts. From these proteins, 19 allergenic proteins in mealworm meal were identified from the 161 unique peptides that were generated after Alcalase^®^ or pepsin in vitro digestion of the mealworm proteins. The coverage and TSC ranged from 3 to 70% and 0 to 106, whatever the conditions (control, pretreatment, and simultaneous) and enzyme (Alcalase^®^ and pepsin). The 19 allergenic proteins were identified according to the publication of Barre et al. in 2018 [35]. The majority of protein MWs ranged from 11 to 51 kDa (~74%), but five of the identified proteins ranged from 84 to 123 kDa. Most of the identified proteins had good peptide coverage, with ~31% of proteins having >10% of the sequence coverage and ~31% of proteins having >20% sequence coverage.

Globally, the in vitro digestion of mealworm allergenic proteins by pepsin was more efficient than Alcalase^®^, since higher TSC values were obtained for all 19 allergenic proteins (Table 1). Regardless of the condition tested (control, pretreated, and simultaneous), allergenic proteins #5, 8, 14, 15, 16, and 17 were more susceptible to in vitro digestion by Alcalase^®^, as illustrated by high TSC values (TSC>10). Specifically, allergenic proteins #1, 2, and 6 were more resistant to hydrolysis by Alcalase^®^ (TSC<10), whereas proteins #3, 4, 7, 9, 10, 11, 12, 18, and 19 were not digested by this enzyme (TSC values closed to 0). However, some differences were observed in TSC values as a function of the hydrolysis conditions. Indeed, while proteins #1, 2, 6, 8, 14, 18, and 19 were similarly digested by Alcalase^®^, as shown by the TSC detected for all conditions, the in vitro digestion of proteins #5, 13, 15, 16, and 17 was more efficient for the simultaneous treatment than for control and pretreated conditions. Only proteins #4, 9, 10 and 18 were resistant to pepsin hydrolysis, with TSC values < 10, regardless of the hydrolysis conditions (control and pressurization treatments). Protein #1 was not hydrolyzed by any treatment, since similar TSC values were obtained for all conditions. When compared to conventional hydrolysis at atmospheric pressure (0.1 MPa), the application of pressurization treatments (380 MPa for 1 min) before or simultaneous to hydrolysis improved the in vitro digestion of proteins #6, 11, 12, 13, 14, 15, 16, 17, and 19. The hydrolysis of proteins #2, 5, 8, 15, and 19 was enhanced by pretreatment, as compared to control and simultaneous conditions, but the hydrolysis of protein #3 was unaffected.

## 4. Discussion

The purpose of this study was to evaluate the effects of Alcalase^®^ and pepsin hydrolysis under HHP processing conditions on the hydrolysis of allergenic proteins from mealworms. Our results provide the first evidence that HHP-assisted enzymatic hydrolysis by Alcalase^®^ at 380 MPa for 1 min improved the degree of hydrolysis of mealworm proteins at an early stage of hydrolysis as well as in vitro digestion of allergenic proteins. Contrary to Alcalase^®^, HHP-assisted enzymatic hydrolysis by pepsin did not improve the degree of hydrolysis of mealworm proteins. However, the high pressure used as pretreatment condition enhanced the in vitro digestion of allergenic proteins by pepsin.

### 4.1. Effects of High Hydrostatic Pressure-Assisted Enzymatic Hydrolysis on Protein Degradation and Degree of Hydrolysis

Our results showed that Alcalase^®^ hydrolysis of mealworm proteins under HHP (simultaneous condition-380 MPa for 1 min) improved the degree of hydrolysis by 24 and 22% after 60 min of hydrolysis when compared to the control and pretreated conditions, respectively (Figure 2), despite no major changes being observed in terms of protein degradation during hydrolysis (Figure 1). This non-correlation between SDS-PAGE profiles and degree of hydrolysis results could be explained by the generation of very low molecular weight peptides (largely < to 10 kDa), which could not be detected due to their migration outside the gel. Consequently, and specifically regarding the degree of hydrolysis, the result indicates that HHP treatment might have facilitated the mealworm protein conformational changes that are needed to increase the effectiveness of enzymatic digestion by providing Alcalase^®^ access to the buried cleavage site at the very beginning of hydrolysis (until 10 min). Previous studies using various enzymes demonstrated that HHP-assisted enzymatic hydrolysis induced the exposure of new cleavage sites through protein unfolding, which enhanced enzyme activity, reduced hydrolysis time [36], and improved the degree of hydrolysis and concentration of peptides generated in protein hydrolysates [37,38,39]. In addition, the DH of mealworm proteins obtained was significantly increased (*p* < 0.05) during HHP-assisted enzymatic hydrolysis by Alcalase^®^. This increase was drastically improved as compared to the control (0.1 MPa) (Figure 2A), but was lower than similar studies [38,40,41], where DH increases ranged from 17% to 58% for pressure-treated protein hydrolysates as compared to the control (0.1 MPa). The effectiveness of HHP treatment on the enzymatic hydrolysis depends on specific parameters, such as substrate/enzyme ratio, pressure level, and treatment duration. Consequently, different hypotheses could explain the lack of influence of HHP on the in vitro digestion of mealworm protein. First, the duration of HHP treatment (1 min) was too short to induce optimal unfolding of mealworm proteins to improve Alcalase^®^ access to buried sites. Second, irreversible protein aggregation occurring during the commercial-scale production of mealworm meals could decrease the efficiency of HHP and enzymatic hydrolysis. Indeed, the mealworm powders that were used for this study were roasted at approximately 107 °C and ground from fresh larvae [42]. When using the same mealworm powder from Entomo Farm, Stone et al. (2019) observed that the heating step of the insects during processing could result in protein denaturation, exposing hydrophobic groups and leading to protein aggregation [42]. Womeni et al. [43] demonstrated that roasting and grinding enhanced the aggregation of proteins, which makes the insect products unsuitable for food formulations due to their low solubility. A recent study published by Kröncke et al. [44] confirmed that oven drying of *T. molitor* larvae decreased the quality of proteins and reduced their solubility by 74% [44]. Consequently, the large amount of protein aggregates generated during production of mealworm meal rendered HHP ineffective for unfolding the proteins, leaving certain bonds in *T. molitor* proteins buried and inaccessible to Alcalase^®^, which decreased the proteolysis efficiency [45,46]. This second hypothesis is especially appealing given the large MW protein aggregates that were observed in the sodium dodecyl sulfate-polyacrylamide gel electrophoresis (SDS-PAGE) wells for control, pretreated, and simultaneous Alcalase^®^ experimental conditions, and could explain why no significant DH was observed between the control and pretreatment conditions. Contrary to Alcalase^®^ hydrolysis, coupling HHP and pepsin had no impact on the DH of mealworm proteins, despite the differences of protein degradation profiles observed in Figure 1. As mentioned for Alcalase^®^, it is difficult to correlate the results obtained in Figure 1; Figure 2, since very low molecular weight peptides could not be not detected in electrophoresis gels. The short duration of HHP treatment and the presence of protein aggregates in mealworm meal could explain the inability of HHP to improve protein digestion in vitro, as hypothesized for Alcalase^®^. Cleavage specificities are also important, since Alcalase^®^ has broad specificity, hydrolyzing most peptide bonds. It preferentially hydrolyzes those containing aromatic amino acid residues whereas pepsin is more specific and cleaves peptide bonds following Phe or Tyr residues, as well as other hydrophobic amino acids. The recent study of Dai et al. [47] confirmed the importance of enzyme specificity during hydrolysis of *T. molitor* larva protein, since Alcalase^®^ was the most efficient enzyme in terms of degree of hydrolysis compared to various other commercial enzymes (trypsin, Neutrase, papain, and pepsin) [47].

### 4.2. Digestion of Mealworm Allergenic Proteins by Pressurization Treatments

Recent publications demonstrated cross-reactivity between edible insects and other Arthropoda (crustaceans, mite), identifying different proteins that are involved in muscle contraction (actin, myosin, tropomyosin, troponin T and C, tubulin), in enzymatic pathways (arginine kinase 1, alpha-amylase) or part of the hemolymphatic system (hexamerin 1 and 2) as pan-allergens [48]. Globally, the results that are presented in Table 1 showed that pepsin was more efficient than Alcalase^®^ for hydrolysis of allergenic mealworm proteins, despite the fact that, on the whole, the DH that was obtained with Alcalase^®^ was higher (Figure 2). Our results also demonstrated that HHP, applied as a pretreatment before in vitro digestion or simultaneously with enzymatic hydrolysis, improved the in vitro digestion of specific mealworm allergenic proteins. These results are consistent with previous publications evaluating the effects of HHP coupled to enzymatic hydrolysis on the potential allergenicity of major protein allergens from different food matrices [49,50,51,52]. Indeed, after HHP-assisted enzymatic hydrolysis, these protein hydrolysates exhibited non-antigenic properties that were superior to those of proteins only treated with enzymatic hydrolysis. The production of hydrolysates with lower immunoreactivity from pressure-treated native protein is induced by increasing the protein susceptibility to enzymatic action by exposing new cleavage sites that allow for the proteases to reach otherwise buried hydrolysis sites.

However, our work also showed that HHP-assisted enzymatic hydrolysis had a limited effect on tropomyosin, myosin heavy chain, troponin, and tubulin proteolysis, as measured by TSC and enzyme efficiency when compared to the control condition. In the literature, the efficiency of Alcalase^®^ and pepsin for the hydrolysis of these specific insect allergenic proteins has been scarcely reported. Tropomyosin from shrimp, being classed as arthropods, was sensitive to HHP, since pressurization at 500 MPa for 10 min combined with thermal treatment at 55 °C decreased the protein allergenicity by 73.59% as compared to a boiling treatment [52]. However, tropomyosin is usually reported as heat stable and resistant to gastrointestinal digestion [53]. More specifically, it was demonstrated that pepsin could only slightly hydrolyze oyster tropomyosin, which demonstrated that tropomyosin has relatively good resistance to this enzyme. Nevertheless, Mejrhit et al. [54] found that shrimp tropomyosin IgE binding was decreased after heat and pepsin treatments. Hall, Johnson, and Liceaga [19] demonstrated that Alcalase^®^ hydrolysis of cricket protein changed the binding characteristics of cricket tropomyosin to IgE, which indicated the susceptibility of tropomyosin to Alcalase^®^ proteolysis. Similar results were obtained using house cricket *Acheta domesticus*, desert locust *Schistocerca gregaria* and yellow mealworm *T. molitor* [4]. To the best of our knowledge, there are no previous reports on the impact of Alcalase^®^ or pepsin hydrolysis, coupled or not to HHP, on the vitro digestion of myosin heavy chain, troponin and tubulin from edible insects. However, Deng et al. [55] reported that pepsin was efficient for the proteolysis of myosin heavy chain and troponin from shrimp, while Alcalase^®^ was often used for the hydrolysis of muscle proteins from meat and fish-based products [56]. Consequently, the low number of TSC for muscular mealworm proteins after enzymatic hydrolysis or the similar number of TSC obtained for control and pressurization conditions can be explained by irreversible protein aggregation induced by HHP, which drastically decreased the proteolytic activity of both Alcalase^®^ and pepsin used at atmospheric pressure or in combination with HHP.

## 5. Conclusions

This preliminary study demonstrated that HHP-assisted enzymatic hydrolysis by Alcalase^®^ improved the degree of hydrolysis of mealworm protein at the very beginning of digestion, despite no modification of protein degradation profiles. Moreover, pressurization treatments that were coupled to hydrolysis by Alcalase^®^ and pepsin further increased the in vitro digestion of specific allergenic proteins. However, the presence of protein aggregates in the initial mealworm meal powder decreased the efficiency of enzymatic hydrolysis and pressurization treatment. Nevertheless, this work has produced encouraging results by combining HHP and enzymatic hydrolysis for the proteolysis of allergenic mealworm proteins. Besides, experiments are currently under way in order to understand the impact of HHP parameters on the denaturation and aggregation of edible insect proteins. Further research on native mealworm proteins extracted from fresh larvae and subject to minimal heat treatment is necessary to improve the combination of HHP and enzymatic hydrolysis.

## Figures and Tables

**Table 1:** Mealworm allergenic protein precursors from peptides generated after Alcalase^®^ and pepsin in vitro digestion of mealworm proteins.

Protein #	Identified Proteins *	MW (kDa)	UniProt ID	Number of Unique Peptides	Coverage (%)	Total Spectrum Count (TSC) ^**^					
						Alcalase^®^			Pepsin		
						C ^1^	P ^2^	S ^3^	C	P	S
1	Myosin heavy chain	123	A0A139WDZ4	15	7	2	4	4	21	31	19
2	Myosin heavy chain	141	A0A139WE70	8	6	2	4	6	9	22	10
3	Myosin heavy chain	103	A0A139WE10	2	9	0	0	0	20	0	21
4	Tropomyosin-1	40	A0A139WAN8	3	9	0	0	0	3	2	1
5	Actin-87E	42	D6WF19	28	39	24	31	51	42	106	71
6	Hexamerin 2	84	A0A288EPS5	7	9	4	6	7	11	28	22
7	Hexamerin 1	86	A0A288EIN5	7	11	0	0	8	7	13	11
8	Arginine kinase 1	40	A0A139WNX9	7	17	10	14	12	9	28	18
9	Troponin T	46	D6W953	3	3	0	1	0	3	6	7
10	Troponin C	17	D6WZP8	1	7	1	1	0	1	2	1
11	Tubulin beta chain	50	D6WSV2	10	16	1	1	1	10	15	17
12	Tubulin alpha chain	50	D6WBN7	5	13	1	0	1	5	10	10
13	Alpha-amylase	51	P56634	6	13	2	1	7	6	21	15
14	Larval cuticle protein A2B	12	P80682	21	70	13	10	16	23	58	52
15	Larval cuticle protein F1	15	Q9TXD9	13	48	13	9	27	18	42	26
16	Larval cuticle protein A1A	18	P80681	5	33	6	6	18	14	58	54
17	Larval cuticle protein A3A	14	P80683	8	58	16	9	21	17	55	59
18	Larval cuticle protein 8	11	D6WMB1	2	15	1	0	4	4	6	2
19	Larval / pupal cuticle protein H1C	21	P80686	10	33	0	0	2	14	29	19

* The probability of protein identification was over 95%. ** The peptide identification probability was ranging from 96 to 100%. ^1^ Control, ^2^ Pretreated, and ^3^ Simultaneous conditions.
